# Genetic epidemiology of infectious diseases in humans: design of population-based studies.

**DOI:** 10.3201/eid0404.980409

**Published:** 1998

**Authors:** L Abel, A J Dessein

**Affiliations:** Institut National de la Santé et de la Recherche Médicale Unit 436, Mathematical and Statistical Modeling in Biology and Medicine, CHU Pitié-Salpêtrière, Paris, France. abel@biomath.jussieu.fr

## Abstract

The spread and clinical manifestations of an infection in human populations depend on a variety of factors, among them host genetics. Familial linkage studies used in genetic epidemiology to identify host genes test for nonrandom segregation of a trait with a few candidate chromosomal regions or any regions in the genome (genomewide search). When a clear major gene model can be inferred and reliable epidemiologic information is collected (e.g., in schistosomiasis), parametric linkage studies are used. When the genetic model cannot be defined (e.g., in leprosy and malaria), nonparametric linkage studies (e.g., sibling-pair studies) are recommended. Once evidence of linkage is obtained, the gene can be identified by polymorphisms strongly associated with the trait. When the tested polymorphism is in strong linkage disequilibrium with the disease allele or is the disease allele itself (e.g., in HIV infection and malaria), association studies can directly identify the disease gene. Finally, the role of the detected polymorphism in causing the trait is validated by functional studies.


### Genetic Epidemiology of Infectious Diseases in Humans: Design of Population-Based Studies

**Laurent Abel* and Alain J. Dessein†**
*Institut National de la Santé et de la Recherche Médicale Unit 436, Paris, France; and †Institut National de la Santé et de la Recherche Médicale Unit 399, Marseille, France

The spread and clinical manifestations of an infection in human populations depend on a variety of factors, among them host genetics. Familial linkage studies used in genetic epidemiology to identify host genes test for nonrandom segregation of a trait with a few candidate chromosomal regions or any regions in the genome (genomewide search). When a clear major gene model can be inferred and reliable epidemiologic information is collected (e.g., in schistosomiasis), parametric linkage studies are used. When the genetic model cannot be defined (e.g., in leprosy and malaria), nonparametric linkage studies (e.g., sibling-pair studies) are recommended. Once evidence of linkage is obtained, the gene can be identified by polymorphisms strongly associated with the trait. When the tested polymorphism is in strong linkage disequilibrium with the disease allele or is the disease allele itself (e.g., in HIV infection and malaria), association studies can directly identify the disease gene. Finally, the role of the detected polymorphism in causing the trait is validated by functional studies.

The profound influence of the host's genetic makeup on resistance to infections has been established in numerous animal studies (1,2) in which disease phenotypes, environmental factors, and crosses can be controlled. Furthermore, recent developments (e.g., use of gene knockout or mutant and transgenic mice) allow genetic analysis of complex traits involved in susceptibility or resistance to infectious pathogens (2,3). As a result of these new developments, the *Lsh/Ity/Bcg* gene was isolated on mouse chromosome 1, which controls innate early susceptibility to several *Mycobacterium* species, as well as other intracellular pathogens (e.g., *Salmonella* Typhimurium, *Leishmania donovani*) (2,4), and was further identified and designated natural resistance-associated macrophage protein 1 (Nramp1) (5). Involvement of a gene in an experimental infection does not imply that differences in susceptibility or resistance to that infection in human populations can be accounted for by polymorphisms in the human homologue of this gene. Genetic epidemiology studies (6,7) combine epidemiologic and genetic information to identify the genes that influence substantially the expression of human complex phenotypes, such as infectious disease-related traits. Epidemiologic information includes measured risk factors that could influence the trait under study (e.g., contamination by the infectious agent, age). Genetic information is derived from familial relationships between study participants (collection of families) or from the typing of genetic markers. Recent maps of the human genome established on the basis of highly polymorphic markers (8) are a fundamental tool for studies involving genetic markers, and two strategies can be used in this context. The first, the candidate gene method, is the typing of a few markers in a limited number of chromosomal regions containing genes related to the phenotype under study. The second is a random search along the whole genome (genomewide search) for chromosomal regions that could be involved in the control of the phenotype.

Address for correspondence: Laurent Abel, INSERM U.436, Mathematical and Statistical Modeling in Biology and Medicine, CHU Pitié-Salpêtrière, 91 Bd de l'Hôpital, 75013 Paris, France; fax 33-1-45-85-15-29; e-mail: abel@biomath.jussieu.fr.

The genetic epidemiology of human infectious diseases differs from the genetic study of other complex phenotypes in three ways. 1) Environmental factors influencing the risk for infection are generally known and when accurately measured, can be included in the analysis; 2) Choice of candidate genes is strongly determined by the gene's function and response to the studied pathogen or by mouse-human chromosome tests that exploit the identification of murine resistance loci; and 3) Major genes involved in the response to a given pathogen can be identified by characterizing phenotypic response to pathogen exposure, such as clinical response, biologic response (intensity of infection), and immunologic response (levels of antibodies or cytokines). The role of genetic factors in the control of these phenotypic responses is generally suggested by twin studies, by strong ethnic differences, or by the great variability of individual phenotypes within their familial aggregation. Specific statistical methods are used to identify these genetic factors and to distinguish them from environmental factors causing the familial resemblance. All these statistical methods search for one or more genes that influence the studied phenotype and are classically divided into parametric and nonparametric. Parametric, or model-based, methods (segregation analysis and linkage analysis by the classical lod-score method) require defining the model and specifying the relationship between the phenotype and factors (mainly a putative gene and environmental covariates) that may influence its expression. Nonparametric or model-free methods (nonparametric linkage analysis and association studies) study the genetic factors influencing a phenotype without specifying the model. Each method has advantages and disadvantages; however, the two methods complement each other. The choice of a design for a particular study depends on several factors related to the phenotype (e.g., nature, frequency), population, accurate measurement of environmental factors, and known genetic background. Both methods have led to successful gene localizations and identifications in the analysis of several infectious disease phenotypes (9,10).

### Parametric (Model-Based) Studies

Parametric studies require explicit specification of the model, i.e., the definition of the relationship between the observed phenotype and the putative genotype. In a simple monogenic disease due to a diallelic gene (D,d), the model is specified by the frequency of the deleterious allele (D for example) and the three probabilities for a person to have the disease, given the presence of genotype DD, Dd, or dd (penetrances). For complex instances, such as susceptibility/resistance, the susceptibility (or the resistance) depends not only on a putative genotype but also on environmental factors that may influence exposure. In such cases, the phenotype/genotype model includes, in addition to the frequency of the deleterious allele, all the parameters that describe and quantify the relationship between susceptibility and the relevant genetic and environmental factors. This relationship can be mathematically expressed in several ways, most recently regression methods that define model parameters in terms of regression coefficients. Furthermore, regression methods could be used to analyze binary (11) as well as quantitative (12) phenotypes. In quantitative phenotypes, the effect of a genotype is defined in terms of three different phenotypic means depending on the genotypes of the study participants. Parametric methods are based on two kinds of complementary analyses, segregation analysis and linkage analysis by the classical lod-score method (13). Both require epidemiologic information (i.e., the measure of the phenotype and of all relevant environmental factors) for each family member. Linkage analysis needs the typing of genetic markers.

### Parametric Segregation Analysis

Segregation analysis is the first step in determining from family data how a given phenotype was inherited. Familial aggregation of infection-related phenotypes can result from genetic relationships, shared environment, and cultural habits. The goal of segregation analysis is to discriminate between these factors, primarily to test for the existence of a single gene, called a major gene. The major gene is not the only gene involved in the expression of the phenotype; rather, of all involved genes, this one has an effect important enough to distinguish it from the others. For a binary clinical phenotype (affected/unaffected by the disease), this effect can be expressed in terms of relative risks, e.g., the ratio of the probability for being infected given a DD genotype to the probability of being infected given a dd genotype. For a quantitative phenotype, this effect is measured by the proportion of the phenotypic variance explained by the major gene (heritability due to the gene). Primarily, segregation analysis uses maximum likelihood methods to test whether the observed familial distributions of the phenotype fit the distributions expected under different hypotheses of familial transmission (in particular the segregation of a major gene). When evidence indicates a major gene, segregation analysis estimates the measurements for the phenotype/genotype model, which are required for parametric linkage analysis.

### Parametric Linkage Analysis

Linkage analysis by the classical lod-score method (13) confirms and locates the gene, detected by segregation analysis (denoted as the phenotype locus). Linkage analysis tests whether, in families, the phenotype locus is transmitted with genetic markers of known chromosomal location. The lod score is a likelihood ratio testing the hypothesis of linkage (against the hypothesis of no linkage) for different genetic distances (or recombination fractions) between the phenotype locus and the marker locus (14). Classically, two conclusions can be reached with a lod-score analysis: 1) linkage between the two loci when the lod score is above a given threshold, and 2) exclusion of linkage between the two loci when the lod score is below a given threshold. Linkage with the phenotype locus can be tested marker by marker (two-point analysis) or by a set of linked markers (multipoint analysis). In linkage, as in segregation analysis, all inferences for individual genotypes at the phenotype locus are made from individual phenotypes and the specified phenotype/genotype model; the lod-score method is most powerful when this model is well defined. A mispecification of the phenotype/genotype model, however, can lead to both inability to detect linkage (and therefore to false exclusion of the region containing the phenotype locus) and to a bias in the recombination fraction estimate (i.e., the genetic distance) between the phenotype locus and the marker locus (15). Nevertheless, such a mispecification does not affect the robustness of the method; i.e., it does not lead to false conclusions in favor of linkage, as long as only one phenotype/genotype model is tested. Correction for multiple testing should accompany the use of several phenotype/genotype models. Similar problems occur when several markers are tested, and guidelines have been proposed to adapt lod-score thresholds to the context of genomewide search (16). Another problem arises when marker data are missing for some family members. In this case, linkage analysis also depends on marker allele frequencies; mispecification of these frequencies can affect both the power and robustness of the method. Multiple marker testing and mispecification of marker allele frequencies are also common problems to the nonparametric methods.

### Model-Based Studies and Infectious Diseases

### Leprosy Studies

Several segregation analyses have been performed in infectious diseases; some suggest that a recessive major gene may play a role in leprosy subtypes (lepromatous or nonlepromatous) (17-19). A recessive major gene was also found to influence leprosy regardless of the clinical defined subtype, in pedigrees of large families from a small Caribbean island (17); the frequency of the deleterious allele was estimated to be 0.3 (9% of homozygous persons predisposed to leprosy); by age 60, the penetrance was approximately 0.6 for predisposed homozygous, whereas it remained below 0.02 for others. Lod-score analysis could not find any linkage between this leprosy susceptibility locus and five markers (including HLA) that were typed in this population (20).

### Malaria Studies

In malaria, segregation analyses have focused on a quantitative phenotype measuring the intensity of infection, i.e., parasitemia levels. Although one study showed the role of a recessive major gene controlling levels of parasitemia (21), two subsequent studies found evidence of a more complex genetic mechanism (22,23). The discrepancies in these results can be explained by several factors related to the host, the parasite, and mosquito transmission. However, all studies showed correlations between siblings and between age and infection (children becoming more often infected than adults). Further genetic analyses such as sibling-pair (sib-pair) study designs should focus on infection in young children.

### Schistosomiasis Studies

Model-based studies have been particularly successful in finding susceptibility genes in schistosomiasis. Several reports indicated that infection intensity was largely determined by the susceptibility/resistance of infected persons (24). In a Brazilian population, segregation analysis showed that the intensity of infection by *Schistosoma mansoni* was controlled by a major gene (25). This gene, SM1, accounts for 66% of the infection intensity variance that remains after other covariate effects (water contact levels, age, gender) have been taken into account. Under this major gene model, approximately 3% of the population is homozygous and predisposed to very high infection levels, 68% is homozygous resistant, and 29% is heterozygous with intermediate levels of resistance (Figure 1). Parametric linkage analysis using the model estimated from segregation analysis was used to locate the gene. A genomewide search was carried out, and SM1 was mapped to human chromosome 5q31-q33, a genetic region that contains several genes encoding molecules that control T-lymphocyte differentiation (26). More recently, a study in a Senegalese population confirmed the presence of a locus influencing *S. mansoni* infection levels on chromosome 5q31-q33 (27). Furthermore, this region has been linked with loci related to

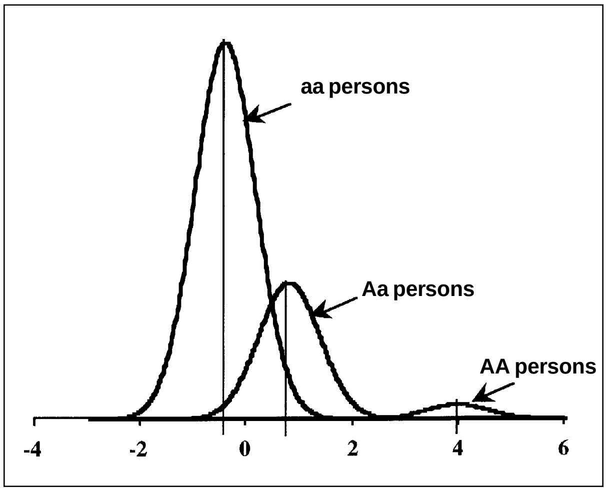

Figure 1. Distribution of the adjusted standardized infection intensities by *Schistosoma mansoni* predicted by the major gene model obtained from segregation analysis and used for linkage analysis. The frequency of allele A predisposing to high infection levels was estimated at 0.16 (70% of aa, 27% of Aa, and 3% of AA persons), and the three means (corresponding to vertical lines) were -0.43, 0.78, and 3.96 for aa, Aa, and AA persons, respectively, with a residual variance equal to 0.33.

immunoglobulin E (IgE) and eosinophilia production, i.e., a locus regulating IgE levels (28,29), a locus controlling bronchial hyperresponsiveness in asthma (30), and a locus involved in familial hypereosinophilia (31). This genetic localization, together with observations that human resistance to schistosomiasis is regulated by lymphokines characteristic of Th2 subsets (32) and that resistant homozygotes mount a Th0/2 response while susceptible homozygotes exhibit a Th0/1 response against schistosomes (V. Rodrigues, A. Dessein, unpub. data), argues strongly that differences in human susceptibility to schistosomiasis are influenced by polymorphisms in a gene controlling T-lymphocyte subset differentiation. In this regard, a segregation analysis showed that interleukin 5 (IL-5) levels are also under the control of a major gene in the same Brazilian population used in the study on infection intensity (33), raising the possibility that IL-5 might play a critical role in resistance, a view consistent with the known role of IL-5 in the defense against schistosome infections.

Another trait of interest in schistosomiasis is the phenotype of severe hepatic fibrosis due to *S. mansoni* infection for which the role of genetic factors has been suggested. Segregation analysis conducted in a Sudanese village found evidence of major gene involvement in severe hepatic periportal fibrosis (A. Dessein, L. Abel, unpub. data). Whether this gene and SM1 are one and the same is under investigation.

### Nonparametric (Model-Free) Studies

Nonparametric or model-free studies (nonparametric linkage analysis and association studies) examine the genetic factors influencing a phenotype without specifying the phenotype/genotype model. These studies are strongly recommended when little is known about the relationship between the phenotype and a putative gene as in the study of complex traits (e.g., infectious disease-related traits) when either no segregation analysis has been performed or no clear major gene model can be inferred from segregation analysis. Nonparametric studies test whether or not the alleles of a given marker are distributed at random in persons having a certain phenotypic resemblance. Nonparametric linkage analyses study the distribution of marker alleles inherited from a same ancestor, i.e., alleles identical by descent

(IBD), in persons from the same family (e.g., siblings), whereas association studies examine the distribution of a given marker allele, e.g., HLA-DR2, in persons not from the same family.

### Nonparametric Linkage Analysis

The most commonly used nonparametric linkage analysis is the sib-pair method. Two siblings can share 0, 1, or 2 parental IBD alleles of any locus, and the respective proportions of this sharing under random segregation are simply 0.25, 0.5, and 0.25 (Figure 2). When the phenotype under study is a clinical disease (affected/unaffected), the method tests whether affected sib-pairs share more parental alleles than expected under random segregation. This excess allele sharing can be tested by a simple chi-square, in particular when all parental marker data are known. Maximum likelihood methods have also been developed to analyze data from affected sib-pairs data, such as the maximum likelihood score (34) and a maximum likelihood binomial approach (35), and can lead to more powerful tests. When the phenotypic response under study is quantitative, the method tests whether siblings with close phenotype values share more IBD alleles than siblings with more distant values. This is the basis of the classical approach proposed by Haseman and Elston (36), which regresses the squared difference of the sib-pair phenotypic values on the expected proportion of alleles shared IBD by the sib-pair. Many recent studies have used other methods not detailed here (37-39). Some of these methods are implemented in popular packages, such as MAPMAKER/SIBS (40), which also allow multipoint analysis of sib-pair data. Sib-pair methods have the same problems as parametric linkage analysis with respect to missing parental marker data and testing with multiple markers; in particular, the number of comparisons made influences the significance levels of the tests, and suspected linkage should be confirmed by replication studies. However, affected sib-pair methods have been effective for several diseases, e.g., insulin-dependent diabetes mellitus (41,42), in genomewide searches for human susceptibility genes in a multifactorial phenotype.

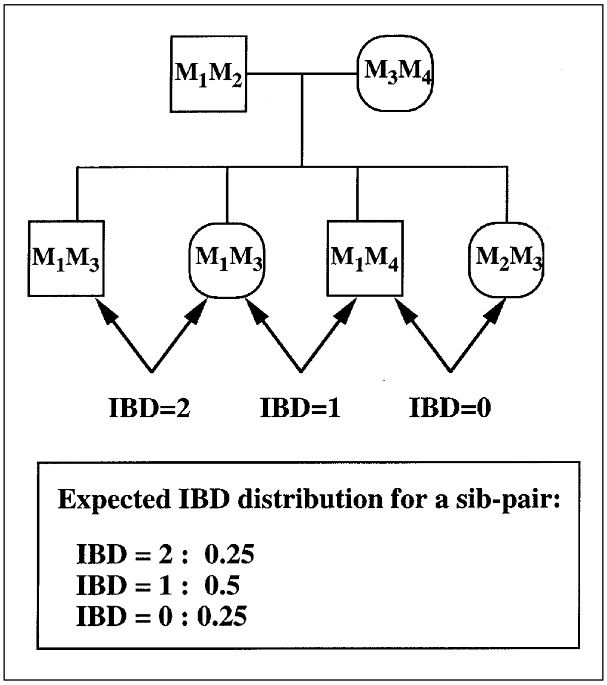

Figure 2. Principle of sib-pair analysis. Two siblings can share 0, 1, or 2 parental marker alleles identical by descent (IBD) at any locus with respective probabilities 0.25, 0.5, and 0.25 under random segregation.

### Leprosy Studies

Sib-pair methods in infectious diseases have focused on candidate regions and have not yet resulted in published genome scans. In leprosy studies using the HLA complex, sib-pair analyses have shown a nonrandom segregation of parental HLA haplotypes in sets of children with tuberculoid leprosy and in siblings with lepromatous leprosy, respectively (18,43,44). However, the observed random segregation of HLA haplotypes in all leprosy patients and in healthy siblings in families with multiple cases of leprosy argued against any involvement of HLA-linked factors in susceptibility to leprosy (44,45). The human gene NRAMP1 (46), homologue of the mouse gene Nramp1, has provided an excellent candidate gene for the study of susceptibility to leprosy. A recent sib-pair study in Vietnam has found linkage between leprosy and NRAMP1 haplotypes consisting of six intragenic variants of NRAMP1 and four polymorphic flanking markers (47) and provided the first evidence that NRAMP1 could be a susceptibility locus for leprosy. Furthermore, this study, combined with segregation analysis performed in the same population (18), suggested genetic heterogeneity according to the ethnic origin of the families (Vietnamese or Chinese), which may explain, at least in part, the results of two previous reports that showed no association between leprosy and distal chromosome 2q where NRAMP1 is located (48,49). Overall, these studies suggest genetic control on at least two levels: a first dependent on non–HLA-linked factors, among which NRAMP1 could play a role, and a second influenced by HLA-linked genes.

### Malaria Studies

Two sib-pair studies focusing on candidate genes have been reported in malaria-related phenotypes. In one (50), nonrandom segregation of the MHC region was found in pairs of dizygous twins with mild clinical malaria. In another (51), the 5q31-q33 region, previously shown to be linked to *S. mansoni* infection levels (26), may be involved in the control of parasitemia due to *Plasmodium falciparum,* although the sample size was too small for definitive conclusion; larger studies are ongoing.

### *Mycobacterium* Studies

The recent demonstration that mutations in the interferon γ receptor 1 (IFNγR1) gene cause disseminated infection due to weakly pathogenic mycobacteria (52,53) was first based on homozygosity mapping (54), a nonparametric linkage method, which locates a rare recessive mutation in consanguineous families by searching for chromosomal regions for which all affected family members are homozygous IBD; i.e., they have received two copies of the same ancestral mutation. In consanguineous infected children from two families, two groups located the genetic defect on chromosome region 6q22-q23 and identified mutations in the IFNγR1 gene leading to the absence of expression of the receptor at the cell surface (52,53). In vitro experiments established the causative relationship between the presence of two mutated IFNγR1 alleles and impaired response to IFN by the cells of these patients (55). Although inherited IFNγR1 deficiency was found in additional families, IFNγR1 mutations were not found in other families with infected patients (J.L. Casanova, pers. comm.), which suggests that other genetic defects may be involved.

### Association Studies

Classic association studies are population-based case-control studies that compare the frequency of a given allele marker in unrelated persons with the phenotype and controls without the phenotype (6,7). G is the disease locus influencing the trait, and M is the marker locus under consideration; G is assumed to be diallelic (D,d) with D being the deleterious allele, and M has several alleles ($M_1$, $M_2$, ..., $M_n$). Association studies examine the role of a particular allele of M. As an example, $M_1$ is said to be associated with the disease under study if it is found at a significantly higher or lower frequency in case-patients than in controls by a simple 2 x 2 contingency table. The simplest explanation for the association is that allele $M_1$ is the deleterious allele D itself. Another explanation is that $M_1$ has no direct effect on the phenotype but is in linkage disequilibrium with allele D. Linkage disequilibrium means two conditions: 1) linkage between locus M and locus G (generally close linkage) and 2) preferential association of allele $M_1$ with allele D; i.e., the $DM_1$ haplotype is more frequent than expected by the respective frequencies of D and $M_1$ (e.g., many present cases are due to one D allele from an ancestor bearing the $DM_1$ haplotype). Even very close linkage alone (only the first condition is fulfilled) does not lead to association, and therefore, the absence of association does not exclude linkage. On the basis of these two explanations, association studies best use the candidate gene approach when they consider markers that are either within or in close linkage with a gene that is related to the phenotypic response. A final explanation for association is the existence of an artifact due to population admixture. For example, a case-control study conducted in a mixture of two subpopulations of which one has a higher disease prevalence and a higher $M_1$ frequency than the second will show a positive association of allele $M_1$ with the disease. To avoid population admixture, family-based association methods have been developed (56), such as the transmission disequilibrium test (TDT) (57). The sampling unit in these methods consists of two parents with an affected child; parental alleles not transmitted to affected children are used as controls. More specifically, the TDT considers affected children of parents heterozygous for $M_1$, e.g., $M_1M_2$, and simply tests whether these children have received $M_1$ with a probability different from 0.5, the value expected under random segregation (Figure 3). The TDT is a very efficient method of detecting the effect of allele $M_1$ when $M_1$ is the deleterious allele D itself

(58). Under this hypothesis that the tested allele $M_1$ is the deleterious allele, TDT was more powerful than even the sib-pair method in the context of a genomewide search involving 500,000 diallelic polymorphisms (5 polymorphisms per gene for an assumed 100,000 genes) (58). However, in the more common situation where $M_1$ is different from D, the power of TDT is highly dependent on the respective frequencies of $M_1$ and D and the strength of the linkage disequilibrium between $M_1$ and D (59). These results indicate that linkage methods are still useful for identifying genes involved in infectious diseases, at least until molecular resources become available for full genomic screening of human genes.

### Leprosy Associations

Most reported associations between leprosy and different HLA alleles could be due to population admixture and statistical problems (multiple testing); therefore, replication studies are very important. In tuberculoid leprosy, the most consistent associations were found with HLA-DR2 (43,45). With HLA molecular typing, a recent study (60) associated Indian tuberculoid leprosy patients and alleles DRB1*1501, DRB1*1502 (both DR2 alleles), and DRB1*1404,

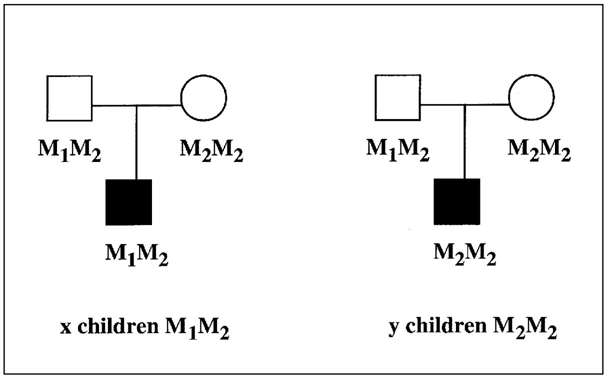

Figure 3. Principle of the transmission disequilibrium test (TDT) for investigating association between a disease and allele $M_1$. The sample consists of x+y families with one affected child and two parents. For ease of presentation, we assume that only one parent is heterozygous for $M_1$ (e.g., $M_1M_2$), although the second parent could be used for the test if he were himself heterozygous for $M_1$. There are x affected children who have received allele $M_1$ from their $M_1M_2$ parent and y who have received $M_2$. The TDT statistic is simply $(x-y)^2/(x+y)$, which is distributed as a chi-square with one degree of freedom.

which are characterized by arginines at position 13 or 70-71. Lepromatous leprosy was associated with HLA-DR3 in several studies (43,45). One report (44) analyzed the transmission of the parental DR3 allele to lepromatous children by a method (similar to TDT) presented several years later (57).

### Malaria Associations

In malaria, population-based association studies have been used to test the hypothesis that certain genetic red cell defects, found more frequently in malaria-endemic areas than in nonendemic-disease areas, had a protective effect against severe malaria (cerebral malaria, severe anemia); the results supported the hypothesis that persons with certain abnormal hemoglobins (61) or glucose-6-phosphate-deshydrogenase deficiency (62) had a reduced risk of developing severe malaria. More recently, a study in Gambia (63) showed that an HLA class I antigen and an HLA class II haplotype were independently associated with protection from severe malaria when a two-stage strategy was used to avoid the problem of multiple testing. In the same population, persons homozygous for a variant of the TNF-α gene promoter, denoted as TNF2, were found to have an increased risk (independent of their HLA alleles) for cerebral malaria (64). A recent work showing that TNF2 is a much stronger transcriptional activator than the more common allele TNF1 (65) indicates that TNF2 affects TNF-α expression and may be directly responsible for the reported association of TNF2 with cerebral malaria. These genetic findings are consistent with immunologic reports showing high TNF-α blood levels in cerebral malaria. Although these genetic polymorphisms (genetic defects of the red cell HLA-TNF polymorphisms) have certainly played a role in selection among populations exposed to malaria infection (61,63), they cannot entirely explain the large interindividual variable responses to the parasite; likely only a minority of genes influencing malaria resistance have been identified (66). This view is supported by a recent report that a coding polymorphism in the intercellular adhesion molecule-1 (ICAM-1), a molecule that affects adherence of infected red blood cells to small vessel endothelium, is associated with an increased susceptibility to cerebral malaria (67).

### HIV Associations

A major advance in the involvement of host factors in HIV-1 infection came when infection status (seropositive/seronegative) was associated with the gene encoding the CC-chemokine receptor 5 (CCR5), the coreceptor of macrophage-tropic HIV-1 strains (68). Two persons exposed many times to HIV-1, yet uninfected, were shown to be homozygous for a defective CCR5 allele containing an internal 32 base-pair deletion (Δ32) (69), and several large cohort studies found HIV-1 infected patients not to be CCR5Δ32 homozygous, whereas exposed HIV-1 seronegative persons did have the defective allele (70-72). Subsequent reports showed that this protection was not complete since some CCR5Δ32 homozygous persons were found to be HIV-1 infected (10). Furthermore, several studies in HIV-1 infected persons found CCR5Δ32 heterozygous status may protect against disease progression (71,72), depending on virus strain (73). However, it is clear that CCR5Δ32 does not alone explain HIV-1 infection status, especially in African populations where Δ32 is absent (70,74), and the search for other host genes involved in susceptibility/resistance to HIV infection will be of major interest.

### Conclusions

Recently developed genetic epidemiology methods and dense human genetic maps, together with the growing availability of candidate genes, are essential for identifying genes that influence human infectious diseases. Nevertheless, investigating the role of genetic factors in a given phenotypic response depends on many different factors related to the phenotype, population, accurate measurement of environmental factors, and previous knowledge; no unique optimal design can be applied for most phenotypic responses related to infectious agents. Among possible study designs, familial linkage studies search for a chromosomal region showing a nonrandom segregation with the phenotype by either focusing on a few candidate regions or using a genomewide search. The main goals of the genome approach are to ensure that all major loci involved in the control of a phenotype are identified and to provide the opportunity to discover new major genes (and consequently physiopathologic pathways) involved in phenotypic responses. Parametric linkage studies are powerful when a clear major gene model can be inferred from segregation analysis. Nonparametric linkage studies are strongly recommended when little is known about the relationship between the studied phenotype and a putative gene, and sib-pair studies have led to successful gene localizations in the analysis of several complex traits, including infectious disease-related traits. Once evidence for linkage is obtained, fine genetic and physical mapping is performed to narrow down the genetic interval. The next step is the search, by molecular methods, of polymorphisms in candidate genes located within the identified interval. These candidate genes are selected from gene databanks or are obtained by a systematic characterization of the genes of the region (positional cloning). On the other hand, association studies performed with candidate genes can directly identify the disease gene when the tested polymorphism is in strong linkage disequilibrium with the disease allele or is the disease allele itself. Finally, evidence for an association should be completed by functional analysis, which will test whether the detected polymorphism modifies the gene expression or the gene product in a manner that can affect susceptibility to the disease.

Progress in the genetic dissection of infectious diseases will also come from the integrated analysis of different phenotypic responses (clinical response, intensity of infection, immunologic response), which can all contribute to the pathologic process, as illustrated in malaria and schistosomiasis studies. The identification of host genes in human infectious diseases will provide new understanding of disease pathogenesis. How this genetic information will modify our approach to prevention and treatment of infectious diseases cannot yet be fully appreciated. However, the identification of susceptibility/resistance genes in schistosomiasis, mycobacterial, and HIV infections has already opened new avenues for the screening of genetically predisposed persons and the development of vaccines.

Dr. Abel is a senior researcher in INSERM (Institut National de la Santé et de la Recherche Médicale) Unit 436, Mathematical and Statistical Modeling in Biology and Medicine, where he heads the group working on the genetic epidemiology of infectious diseases.

Dr. Dessein is professor at the Faculté de Médecine de Marseille-Université de la Méditerranée and head of INSERM Unit 399, Immunology and Genetic of Parasitic Diseases.

### References

1. Wakelin DM, Blackwell JM, editors. Genetics of resistance to bacterial and parasitic infection. London: Taylor and Francis; 1988.
2. McLeod R, Buschman E, Arbuckle LD, Skamene E. Immunogenetics in the analysis of resistance to intracellular pathogens. Curr Opin Immunol 1995;7:539-52.
3. Nadeau JH, Arbuckle LD, Skamene E. Genetic dissection of inflamatory responses. J Inflamm 1995;45:27-48.
4. Blackwell JM, Barton CH, White JK, Roach TIA, Shaw MA, Whitehead SH, et al. Genetic regulation of leishmanial and mycobacterial infections: the Lsh/Ity/Bcg gene story continues. Immunol Lett 1994;43:99-107.
5. Vidal S, Malo D, Vogan K, Skamene E, Gros P. Natural resistance to infection with intracellular parasites: isolation of a candidate for Bcg. Cell 1993;73:469-86.
6. Khoury MJ, Beaty TH, Cohen BH, editors. Fundamentals of Genetic Epidemiology. New York: Oxford University Press; 1993.
7. Lander ES, Schork NJ. Genetic dissection of complex traits. Science 1994;265:2037-48.
8. Dib C, Fauré S, Fizames C, Samson D, Drouot N, Vignal A, et al. A comprehensive genetic map of the human genome based on 5,264 microsatellites. Nature 1996;380:152-4.
9. Hill AVS. Genetics of infectious disease resistance. Curr Opin Genet Dev 1996;6:348-53.
10. Abel L, Dessein AJ. The impact of host genetics on susceptibility to human infectious diseases. Curr Opin Immunol 1997;509-16.
11. Bonney GE. Regressive logistic model for familial disease and other binary traits. Biometrics 1986;42:611-25.
12. Bonney GE. On the statistical determination of major gene mechanisms in continuous human traits: regressive models. Am J Med Genet 1984;18:731-49.
13. Morton NE. Sequential tests for the detection of linkage. Am J Hum Genet 1955;7:277-318.
14. Ott J, editor. Analysis of human genetic linkage. Baltimore and London: The Johns Hopkins University Press; 1991.
15. Clerget-Darpoux F, Bonaïti-Pellié C, Hochez J. Effects of mispecifying genetic parameters in lod-score analysis. Biometrics 1986;42:393-9.
16. Lander E, Kruglyak L. Genetic dissection of complex traits: guidelines for interpreting and reporting linkage results. Nat Genet 1995;11:241-7.
17. Abel L, Demenais F. Detection of major genes for susceptibility to leprosy and its subtypes in a Caribbean island: Desirade. Am J Hum Genet 1988;42:256-66.
18. Abel L, Lap VD, Oberti J, Thuc NV, Cua VV, Guilloud-Bataille M, et al. Complex segregation analysis of leprosy in Vietnam. Genet Epidemiol 1995;12:63-82.
19. Feitosa MF, Borecki I, Krieger H, Beiguelman B, Rao DC. The genetic epidemiology of leprosy in a Brazilian population. Am J Hum Genet 1995;56:1185-95.
20. Abel L, Demenais F, Baule MS, Blanc M, Muller A, Raffoux C, et al. Genetic susceptibility to leprosy on a Caribbean island: linkage analysis with five markers. International Journal of Leprosy 1989;57:465-71.
21. Abel L, Cot M, Mulder L, Carnevale P, Feingold J. Segregation analysis detects a major gene controlling blood infection levels in human malaria. Am J Hum Genet 1992;50:1308-17.
22. Garcia A, Cot M, Chippaux JP, Ranques S, Feingold J, Demenais F, et al. Genetic control of blood infection levels in human malaria: evidence for a complex genetic model. Am J Trop Med Hyg 1998;58:480-8.
23. Rihet P, Abel L, Traoré Y, Traoré-Leroux T, Aucan C, Fumoux F. Human malaria: segregation analysis of blood infection levels in a suburban area and a rural area in Burkina Faso. Genet Epidemiol 1998;15:435-50.
24. Dessein A, Abel L, Couissinier P, Demeure C, Rihet P, Kohlstaedt S, et al. Environmental, genetic and immunological factors in human resistance to *Schistosoma mansoni*. Immunol Invest 1992;21:421-51.
25. Abel L, Demenais F, Prata A, Souza AE, Dessein A. Evidence for the segregation of a major gene in human susceptibility/resistance to infection by Schistosoma mansoni. Am J Hum Genet 1991;48:959-70.
26. Marquet S, Abel L, Hillaire D, Dessein H, Kalil J, Feingold J, et al. Genetic localization of a locus controlling the intensity of infection by *Schistosoma mansoni* on chromosome 5q31-q33. Nat Genet 1996;14:181-4.
27. Müller-Myhsok B, Stelma FF, Guissé-Sow F, Muntau B, Thye T, Burchard GD, et al. Further evidence suggesting the presence of a locus on human chromosome 5q31-q33 influencing the intensity of infection with *Schistosoma mansoni*. Am J Hum Genet 1997;61:452-4.
28. Marsh DG, Neely JD, Breazale DR, Ghosh B, Freidhoff LR, Ehrlich-Kautzky E, et al. Linkage analysis of *IL4* and other chromosome 5q31.1 markers and total serum immunoglobulin E concentrations. Science 1994;264:1152-6.
29. Meyers DA, Postma DS, Panhuysen CIM, Xu J, Amelung PJ, Levitt RC, et al. Evidence for a locus regulating total serum IgE levels mapping to chromosome 5. Genomics 1994;23:464-70.
30. Postma DS, Bleecker ER, Amelung PJ, Holroyd KJ, Xu J, Panhuysen CIM, et al. Genetic susceptibility to asthma—bronchial hyperresponsiveness coinherited with a major gene for atopy. N Engl J Med 1995;333:894-900.
31. Lin AY, Rioux JD, Nutman T, Daly M, Stone V, Nguyen H, et al. A gene for familial hypereosinophilia maps to chromosome 5q31-q33. Am J Hum Genet 1997;Suppl 61:A283.
32. Couissinier-Paris P, Dessein AJ. Schistosoma-specific helper T cell clones from subjects resistant to infection by *Schistosoma mansoni* are Th0/2. Eur J Immunol 1995;25:2295-302.
33. Rodrigues V, Abel L, Piper K, Dessein AJ. Segregation analysis indicates a major gene in the control of interleukine-5 production in humans infected with *Schistosoma mansoni*. Am J Hum Genet 1996;59:453-61.

34. Risch N. Linkage strategies for genetically complex traits. III. The effect of marker polymorphism on analysis of affected relative pairs. Am J Hum Genet 1990;46:242-53.
35. Abel L, Alcais A, Mallet A. Comparison of four sib-pair linkage methods for analyzing sibships with more than two affecteds: interest of the binomial maximum likelihood approach. Genet Epidemiol 1998;15:371-390.
36. Haseman JK, Elston RC. The investigation of linkage between a quantitative trait and a marker locus. Behav Genet 1972;2:3-19.
37. Goldgar DE. Multipoint analysis of human quantitative genetic variation. Am J Hum Genet 1990;47:957-67.
38. Amos CI. Robust variance-components approach for assessing genetic linkage in pedigrees. Am J Hum Genet 1994;54:535-43.
39. Blangero J, Almasy L. Multipoint oligogenic linkage analysis of quantitative traits. Genet Epidemiol 1997;14:959-64.
40. Kruglyak L, Lander ES. Complete multipoint sib-pair analysis of qualitative and quantitative traits. Am J Hum Genet 1995;57:439-54.
41. Davies JL, Kawaguchi Y, Bennett ST, Copeman JB, Cordell HJ, Pritchard LE, et al. A genome-wide search for human type 1 diabetes susceptibility genes. Nature 1994;371:130-6.
42. Hashimoto L, Habita C, Beressi JP, Delepine M, Besse C, Cambon-Thomsen A, et al. Genetic mapping of a suceptibility locus for insulin-dependent diabetes mellitus on chromosome 11q. Nature 1994;371:161-4.
43. van Eden W, de Vries RRP. HLA and leprosy: a reevaluation. Lepr Rev 1984;55:89-104.
44. van Eden W, Gonzalez NM, de Vries RR, Convit J, Van Rood JJ. HLA-linked control of predisposition to lepromatous leprosy. J Infect Dis 1985;151:9-14.
45. Ottenhoff TH, de Vries RR. HLA class II immune response and suppression genes in leprosy. International Journal of Leprosy 1987;55:521-34.
46. Cellier M, Govoni G, Vidal S, Groulx N, Liu J, Sanchez F, et al. Human natural resistance-associated macrophage protein: cDNA cloning, chromosomal mapping, genomic organization, and tissue-specific expression. J Exp Med 1994;180:1741-52.
47. Abel L, Sanchez F, Oberti J, Thuc NV, Hoa LV, Lap VD, et al. Susceptibility to leprosy is linked to the human NRAMP1 gene. J Infect Dis 1998;177:133-45.
48. Shaw MA, Atkinson S, Dockrell H, Hussain R, Lins-Lainson Z, Shaw J, et al. An RFLP map for 2q33-q37 from multicase mycobacterial and leishmanial disease families: no evidence for an Lsh/Ity/Bcg gene homologue influencing susceptibility to leprosy. Ann Hum Genet 1993;57:251-71.
49. Levee G, Liu J, Gicquel B, Chanteau S, Schurr E. Genetic control of susceptibility to leprosy in French Polynesia; no evidence for linkage with markers on telomeric human chromosome 2. International Journal of Leprosy 1994;62:499-511.
50. Jepson A, Sisay-Joof F, Banya W, Hassan-King M, Frodsham A, Bennett S, et al. Genetic linkage of mild malaria to the major histocompatibility complex in Gambian children: study of affected sibling pairs. BMJ 1997;315:96-7.
51. Garcia A, Marquet S, Bucheton B, Hillaire D, Cot M, Fievet N, et al. Linkage analysis of blood *Plasmodium falciparum* levels: interest of the 5q31-q33 region. Am J Trop Med Hyg 1998;58:705-9.
52. Newport MJ, Huxley CM, Huston S, Hawrylowicz CM, Oostra BA, Williamson R, et al. A mutation in the interferon-γ-receptor gene and susceptibility to mycobacterial infection. N Engl J Med 1996;335:1941-9.
53. Jouanguy E, Altare F, Lamhamedi S, Revy P, Emile JF, Newport M, et al. Interferon-γ-receptor deficiency in an infant with fatal Bacille Calmette-Guérin infection. N Engl J Med 1996;335:1956-60.
54. Lander ES, Botstein D. Homozygosity mapping: a way to map human recessive traits with the DNA of inbred children. Science 1987;236:1567-70.
55. Casanova JL, Newport M, Fisher A, Levin M. Inherited interferon gamma receptor deficiency. In: Ochs H, Puck J, Smith C, editors. Primary immunodeficiencies: a molecular and genetic approach. New York: Oxford University Press. In press **1998.**
56. Schaid DJ, Sommer SS. Comparison of statistics for candidate-gene associations using cases and parents. Am J Hum Genet 1994;55:402-9.
57. Spielman RC, McGinnis RE, Ewens WJ. Transmission test for linkage disequilibrium: the insulin gene and insulin-dependent diabetes mellitus (IDDM). Am J Hum Genet 1993;52:506-16.
58. Risch N, Merikangas K. The future of genetic studies of complex human diseases. Science 1996;273:1516-7.
59. Müller-Myhsok B, Abel L. Genetic analysis of complex diseases. Science 1997;275:1328-9.
60. Zerva L, Cizman B, Mehra NK, Alahari SK, Murali R, Zmijewski CM, et al. Arginine at positions 13 or 70-71 in pocket 4 of HLA-DRB1 alleles is associated with susceptibility to tuberculoid leprosy. J Exp Med 1996;183:829-36.
61. Miller LH. Impact of malaria on genetic polymorphism and genetic diseases in Africans and African Americans. Proc Natl Acad Sci U S A 1994;91:2415-9.
62. Ruwende C, Fhoo SC, Snow RW, Yates SNR, Kwiatkowski D, Gupta S, et al. Natural selection of hemi- and heterozygotes for G6PD deficiency in Africa by resistance to severe malaria. Nature 1995;376:246-9.
63. Hill AVS, Allsopp CEM, Kwiatkowski D, Anstey NM, Twumasi P, Rowe PA, et al. Common west African HLA antigens are associated with protection from severe malaria. Nature 1991;352;595-600.
64. McGuire W, Hill AVS, Allsopp CEM, Greenwood BM, Kwiatkowski D. Variation in the TNF-α promoter region associated with susceptibility to cerebral malaria. Nature 1994;371:508-11.
65. Wilson AG, Symons JA, McDowell TL, McDevitt HO, Duff GW. Effects of a polymorphism in the human tumor necrosis factor promoter on transcriptional activation. Proc Natl Acad Sci U S A 1997;94:3195-9.
66. Miller LH. Protective selective pressure. Nature 1996;383:480-1.
67. Fernandez-Reyes D, Craig AG, Kyes SA, Peshu N, Snow RW, Berendt AR, et al. A high frequency African coding polymorphism in the N-terminal domain of ICAM-1 predisposing to cerebral malaria in Kenya. Hum Mol Genet 1997;6:1357-60.

68. Fauci AS. Host factors and the pathogenesis of HIV-induced disease. Nature 1996;384:529-34.
69. Liu R, Paxton WA, Choe S, Ceradini D, Martin SR, Horuk R, et al. Homozygous defect in HIV-1 coreceptor accounts for resistance of some multiply-exposed individuals to HIV-1 infection. Cell 1996;86:367-77.
70. Samson M, Libert F, Doranz BJ, Rucker J, Liesnard C, Farber CM, et al. Resistance to HIV-1 infection in caucasian individuals bearing mutant alleles of the CCR5 chemokine receptor gene. Nature 1996;382:722-5.
71. Dean M, Carrington M, Winkler C, Huttley GA, Smith MW, Allikmets R, et al. Genetic restriction of HIV-1 infection and progression to AIDS by a deletion allele of the CKR5 structural gene. Science 1996;273:1856-61.
72. Huang Y, Paxton WA, Wolinsky SM, Neumann AU, Zhang L, He T, et al. The role of a mutant CCR5 allele in HIV-1 transmission and disease progression. Nature Med 1996;2:1240-3.
73. Michael NL, Chang G, Leslie GL, Mascola JR, Dondero D, Birx DL, et al. The role of viral phenotype and CCR5 gene defects in HIV-1 transmission and disease progression. Nature Med 1997;3:338-40.
74. Martison JJ, Chapman NH, Rees DC, Liu YT, Clagg JB. Global distribution of the CCR5 gene 32-basepair deletion. Nature Genet 1997;16:100-3.
